# Supplementary material for: An mRNA-based T-cell-inducing antigen strengthens COVID-19 vaccine against SARS-CoV-2 variants
Source: Nat Commun. 2023 May 23;14:2962. doi: 10.1038/s41467-023-38751-8 (PMC10204679; doi:10.1038/s41467-023-38751-8)
Supplement: Supplementary file 1 — Supplementary Information [file 41467_2023_38751_MOESM1_ESM.pdf]

Supplementary Materials for

An mRNA-based T-cell-inducing antigen strengthens COVID-19 vaccine against SARS-CoV-2 variants

Wanbo Tai, Shengyong Feng, Benjie Chai, Shuaiyao Lu, Guangyu Zhao, Dong Chen, Wenhai Yu, Liting Ren, Huicheng Shi, Jing Lu, Zhuming Cai, Mujia Pang, Xu Tan, Penghua Wang, Jinzhong Lin, Qiangming Sun, Xiaozhong Peng, Gong Cheng

Corresponding to gongcheng@mail.tsinghua.edu.cn (G.C.); pengxiaozhong@pumc.edu.cn (X.P.); qsun@imbcams.com.cn (Q.S.); linjinzhong@fudan.edu.cn (J.L.).

This file includes:

Supplementary Fig. 1 Physical features and tissue distribution of the LNP system.
Supplementary Fig. 2 Elicitation of memory $CD8^+$ T cells by LNP-*HLA-EPs*.
Supplementary Fig. 3 Immunogenicity of HLA-EPs was not influenced by the mutations in SARS-CoV-2 variants.
Supplementary Fig. 4 Validation of SARS-CoV-2 RBD_{beta} expression.
Supplementary Fig. 5 Humoral immune activation after dual immunization.
Supplementary Fig. 6 Induction of cellular immune response by dual immunization in HLA transgenic mice.
Supplementary Fig. 7 Induction of Th1-biased cellular immune response by dual immunization in macaques.
Supplementary Fig. 8 Clinical and pathological signs in immunized macaques after challenge with SARS-CoV-2.
Supplementary Fig. 9 Viral load in tissues from challenged animals by qRT-PCR.
Supplementary Fig. 10 Uncropped blots for Extended Data Fig. 4b.
Supplementary Table 1 The HLA-I alleles selected for functional epitopes prediction.
Supplementary Table 2 PBMCs donors information.
Supplementary Table 3 Predicted epitopes mutation summary of NSP-$4_{232-444}$ and NSP-6_{1-201}.
Supplementary Table 4 Validated HLA-A*02:01 and HLA-A*11:01 specific peptides in tetramer assay.
Supplementary Table 5 Primers for qRT–PCR.

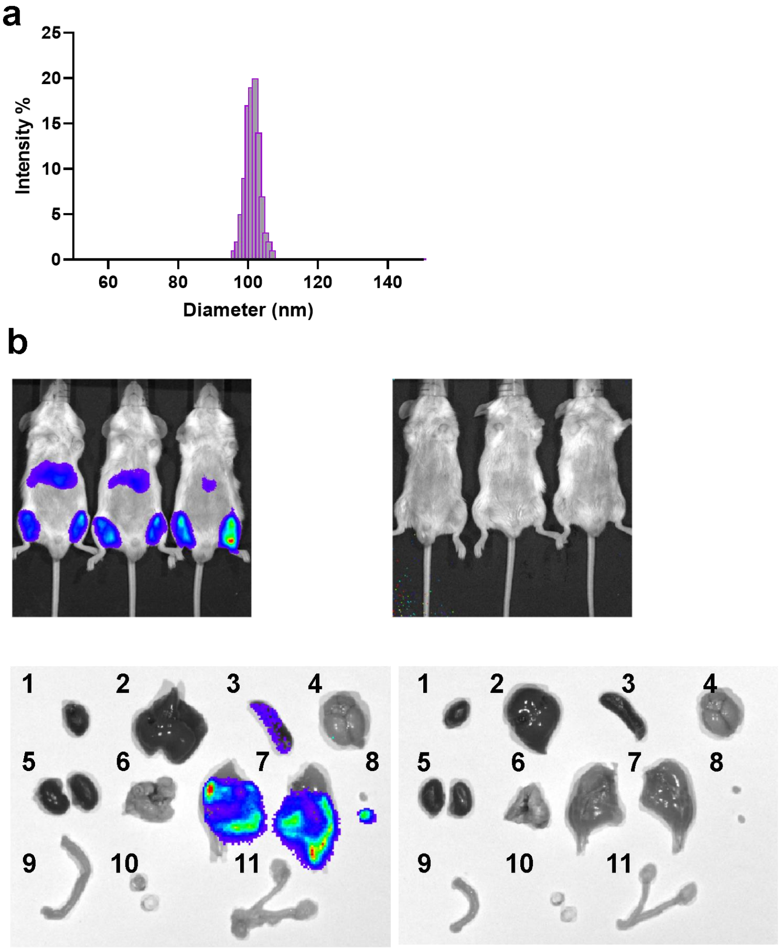

1: Heart; 2: Liver; 3: Spleen; 4: Brain; 5: Kidney; 6: Lung; 7: Muscle;
8: Lymph nodes; 9: Intestine; 10: Eye balls; 11: Ovary

Supplementary Fig. 1 Physical features and tissue distribution of the LNP system.

a, A representative size distribution of the lipid nanoparticles encapsulating mRNA measured by the dynamic light-scattering method. Source data are provided as a Source Data file. **b,** *In vivo* tissue distribution of reporter mRNA-LNPs in mice. Female BALB/c mice were inoculated with 2 μg of luciferase-encoding reporter mRNA-LNPs via intramuscular routes and subjected to *in vivo* imaging system at 6 hours after administration (n = 3), and mice injected with equal-volume PBS were set as control (n = 3).

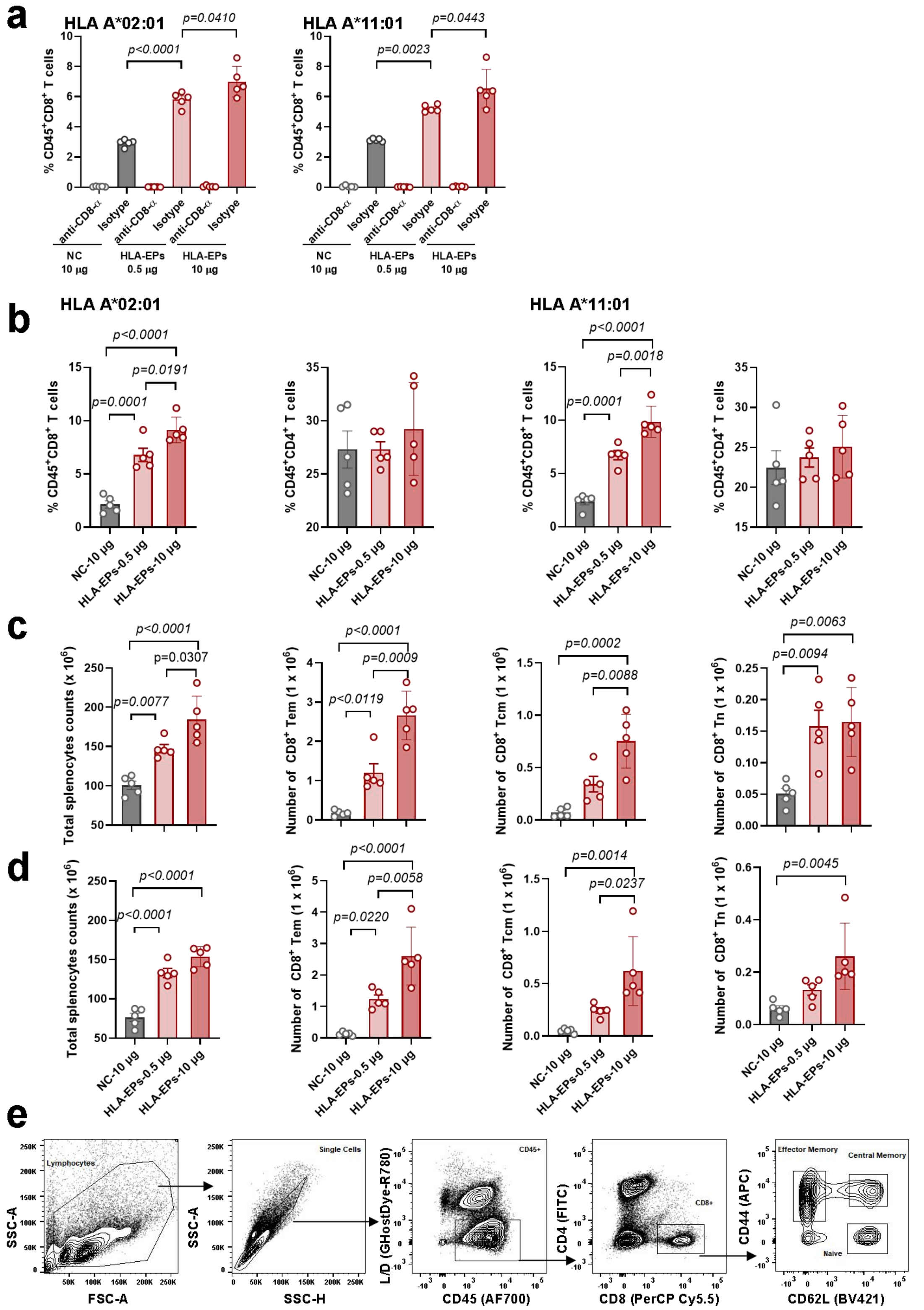

Supplementary Fig. 2 Elicitation of memory CD8$^+$ T cells by LNP-*HLA-EPs*.

Both HLA-A*02:01/DR1 and HLA-A*11:01/DR1 transgenic mice were vaccinated intramuscularly with two doses of LNP-*HLA-EPs* (0.5 µg/dose and 10 µg/dose) or LNP-*NC* (control, 10 µg/dose) with a 3-week interval. At 20 days post-booster immunization, the

immunized mice were injected (i.p.) with an anti-CD8-α antibody (to deplete $CD8^+$ T cells) or IgG2b isotype control antibody (no $CD8^+$ T-cell depletion) (200 mg/mouse). Antibodies were administered again the next day, then blood, lymph nodes and spleens were harvested from the immunized mice (n = 5, HLA-A*02:01/DR1 or HLA-A*11:01/DR1 transgenic mouse) for cell-typing by flow cytometry. **a** The percentages of the lymphocyte subset of $CD45^+CD8^+$ T cells in the PBMCs of immunized HLA-A*02:01/DR1 (**a, left panel**) or HLA-A*11:01/DR1 (**a, right panel**) transgenic mouse injected with an anti-CD8-α or isotype control antibody. **b** The percentages of the lymphocyte subsets of $CD45^+CD8^+$ T cells and $CD45^+CD4^+$ T cells in the spleens of immunized HLA-A*02:01/DR1 (**b, left panel**) or HLA-A*11:01/DR1 transgenic mice (**b, right panel**). **c,d** Total splenocytes, effector memory (Tem) $CD44^+CD62L^-CD8^+$ T cells, central memory (Tcm) $CD44^+CD62L^+CD8^+$ T cells and naïve (Tn) $CD44^-CD62L^+CD8^+$ T cells were analyzed by flow cytometry for immunized HLA-A*02:01/DR1 (**c**) and HLA-A*11:01/DR1 transgenic mice (**d**). The data are presented as the mean ± S.E.M. (n = 5). Source data are provided as a Source Data file. Statistical significance was calculated via one-way ANOVA with Tukey's multiple comparison post-hoc two-sided tests (**a-d**). *P* values were adjusted for multiple comparisons. **e** Gating strategy used in multiparametric flow cytometry analysis.

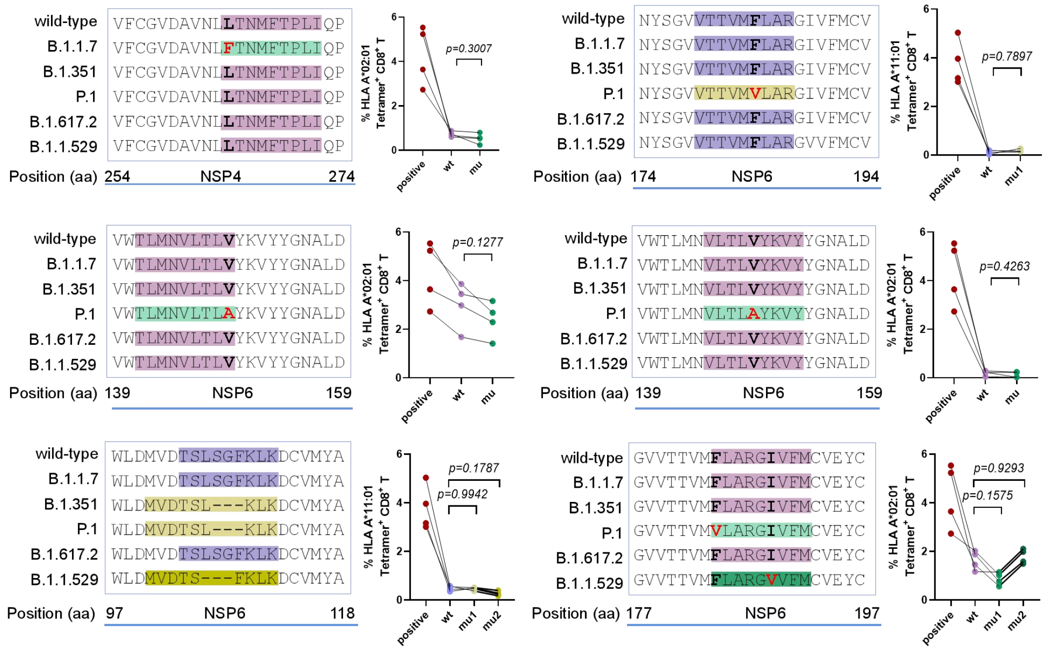

Supplementary Fig. 3 Immunogenicity of HLA-EPs was not influenced by the mutations in SARS-CoV-2 variants.

The peptides covering the predicted wild-type (affinity IC_{50} <200 nM) and mutant HLA-A*02:01/HLA-A*11:01 epitopes within HLA-EPs were synthesized for peptide-specific Tetramer assays. Splenocytes isolated from LNP-*HLA-EP*-immunized HLA-A*02:01/DR1 or HLA-A*11:01/DR1 transgenic mice were harvested and stimulated with the above peptides at 2 μg/ml per peptide, respectively. SARS-CoV-2 originated peptides that were reported to stimulate $CD8^+$ T cell responses (Extended Data Table 3) were used as positive controls. After stimulation, cells were stained with anti-CD45-Alexa Fluor™ 700, CD8-PerCP-Cyanine5.5, and peptide-tetramer-PE mixture. The frequency of HLA-A*02:01- or HLA-A*11:01-tetramer-positive cells was measured by flow cytometry. Data are representative of two independent experiments with three technical replicates. The data were statistically analyzed by one-way ANOVA with Tukey's multiple comparison post-hoc two-sided tests (n = 4). *P* values were adjusted for multiple comparisons. Source data are provided as a Source Data file.

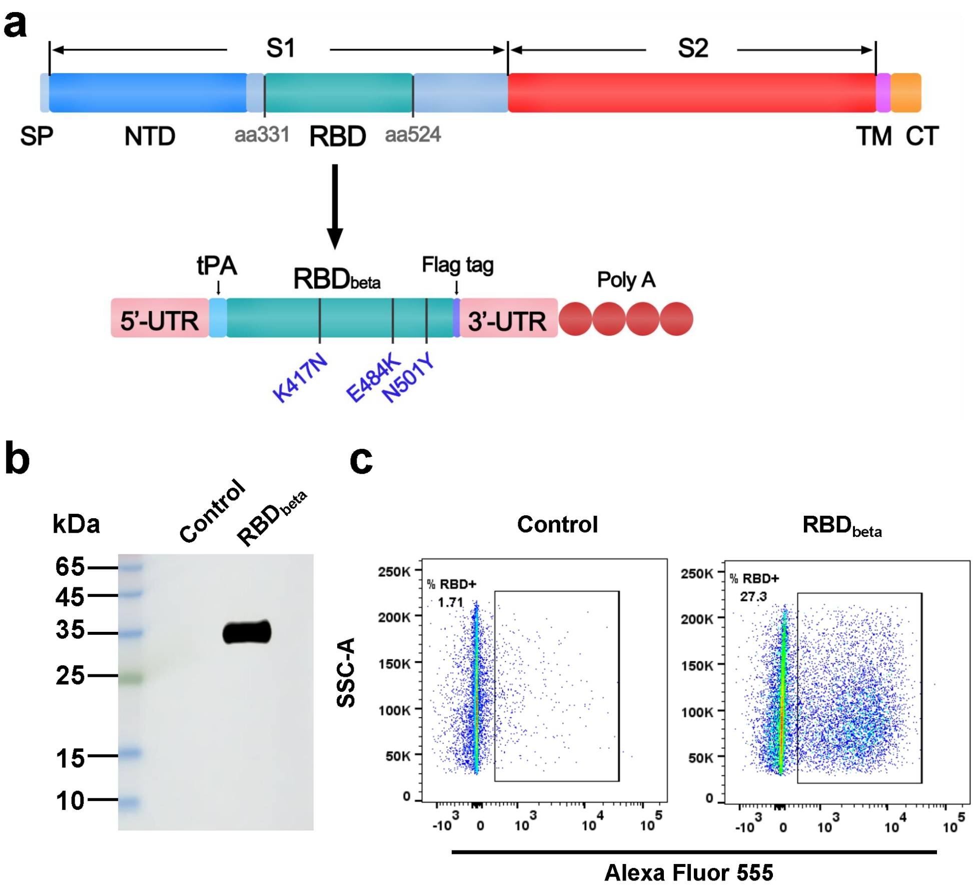

Supplementary Fig. 4 Validation of SARS-CoV-2 RBD$_{beta}$ expression.

a Schematic diagram of the nucleoside-modified SARS-CoV-2 *RBD$_{beta}$* mRNAs. The mRNA is consisted of a 5'-Cap (Cap 1) structure, a 5'-untranslated region (UTR), the nucleoside-modified sequence encoding the SARS-CoV-2 RBD with K417N, E484K and N501Y mutations flanked by a tPA signal peptide and a Flag tag, a 3'-UTR, and a 3'-Poly-A tail. The synthesized nucleoside-modified mRNAs (containing pseudouridines (Ψ) instead of UTPs) were encapsulated with LNPs to form LNP-*RBD$_{beta}$*. **b**,**c** Validation of SARS-CoV-2 RBD$_{beta}$ expression. *RBD$_{beta}$* mRNA was transfected into HEK293T cells, and the supernatants were collected 72 h post-transfection to detect protein expression by Western blotting (**b**). Supernatants from mock cells were used as a negative control. The molecular weights (kDa) of protein markers are listed on the left, and the image is representative of similar results from two independent experiments. The expression of SARS-CoV-2 RBD$_{beta}$ was also validated by the percentage of HEK293T cells with RBD$_{beta}$ positive staining by a flow cytometry assay (**c**), and the images are representative of two independent experiments with three technical replicates.

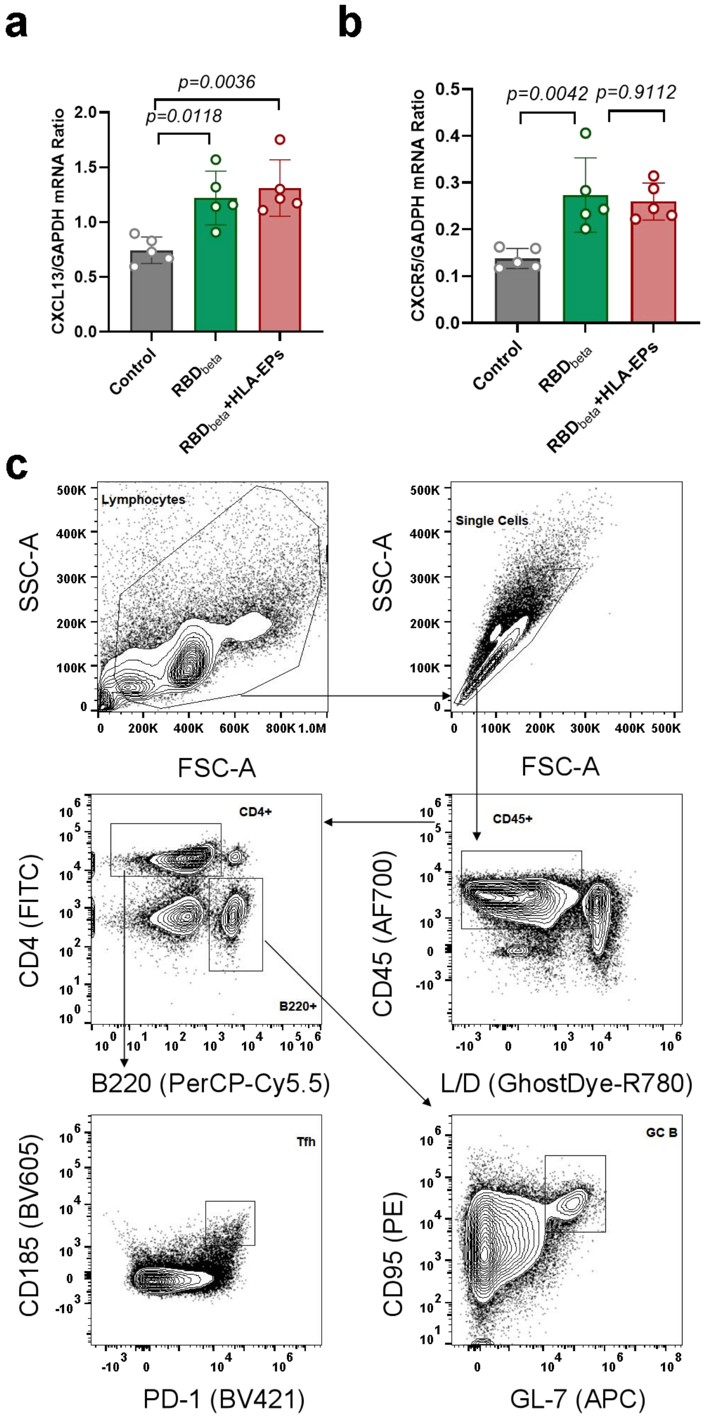

Supplementary Fig. 5 Humoral immune activation after dual immunization. HLA-A*02:01/DR1 transgenic mice (4-5 weeks) were randomly allocated into three groups (n = 5) and vaccinated via the intramuscular route. The same doses were used for a booster immunization 3 weeks post-priming vaccination. The immunized mice were sacrificed at 7 days post-booster vaccination. **a,b** RNA extracted from the lymph nodes was subjected to *CXCR13* (**a**) and *CXCR5* (**b**) mRNA level measurement by qRT–PCR. Data are representative

of two independent experiments with three technical replicates, and all data are presented as the mean ± S.E.M. (n = 5). Adjusted *p* values represent one-way ANOVA with Tukey's multiple comparison post-hoc two-sided tests (**a,b**). Source data are provided as a Source Data file. **c** The lymph nodes were collected from immunized HLA-A*02:01/DR1 transgenic mice (7 days post-second vaccination) for GC B cell ($B220^+CD95^+GL\text{-}7^+$) and Tfh ($CD4^+CD185^+PD\text{-}1^+$) cell analysis by flow cytometry. The gating strategies for GC B and Tfh cells are shown (**c**).

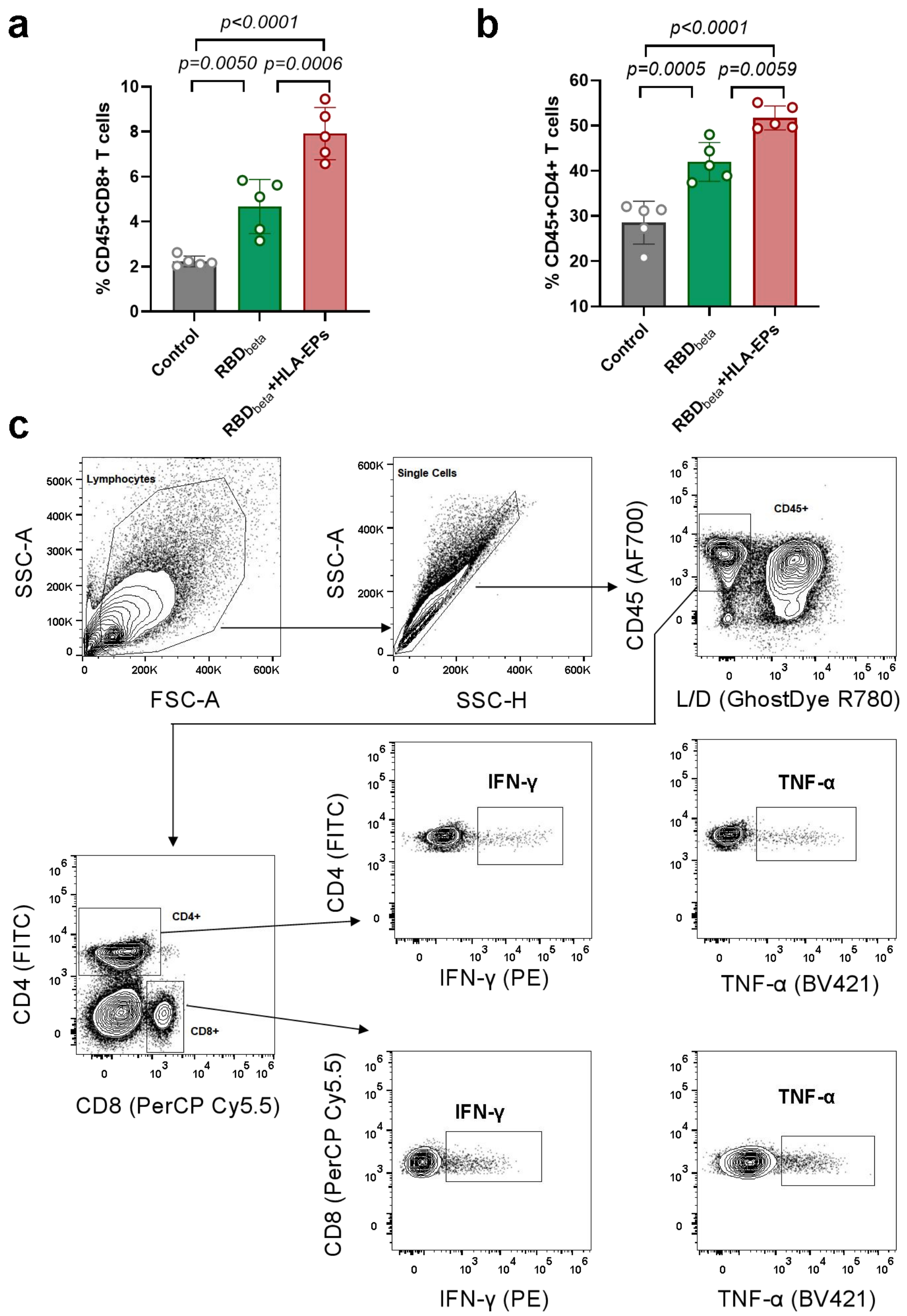

Supplementary Fig. 6 Induction of cellular immune response by dual immunization in HLA transgenic mice.

HLA-A*02:01/DR1 mice (4-5 weeks old) were immunized with PBS, 0.5 µg LNP-RBD_{beta} (0.5 µg per dose), or LNP-RBD_{beta}+LNP-*HLA-EPs* (0.5 µg per dose for either component), and boosted with an equivalent dose 21 days later. The spleens from immunized mice (n = 5, HLA-A*02:01/DR1 transgenic mouse) were harvested on day 21 post the second immunization, and the splenocytes were profiled by flow cytometry. **a,b** The frequencies of the lymphocyte subsets of $CD45^{+}CD8^{+}$ T cells (**a**) and $CD45^{+}CD4^{+}$ T cells (**b**) in immunized

HLA-A*02:01/DR1 transgenic mice. The data are presented as the mean ± S.E.M. (n = 5). Adjusted *p* values represent the results of one-way ANOVA with Tukey's multiple comparison post-hoc two-sided tests of the immunized groups. Source data are provided as a Source Data file. **c** Gating strategy used for intracellular cytokine staining by multiparametric flow cytometry analysis.

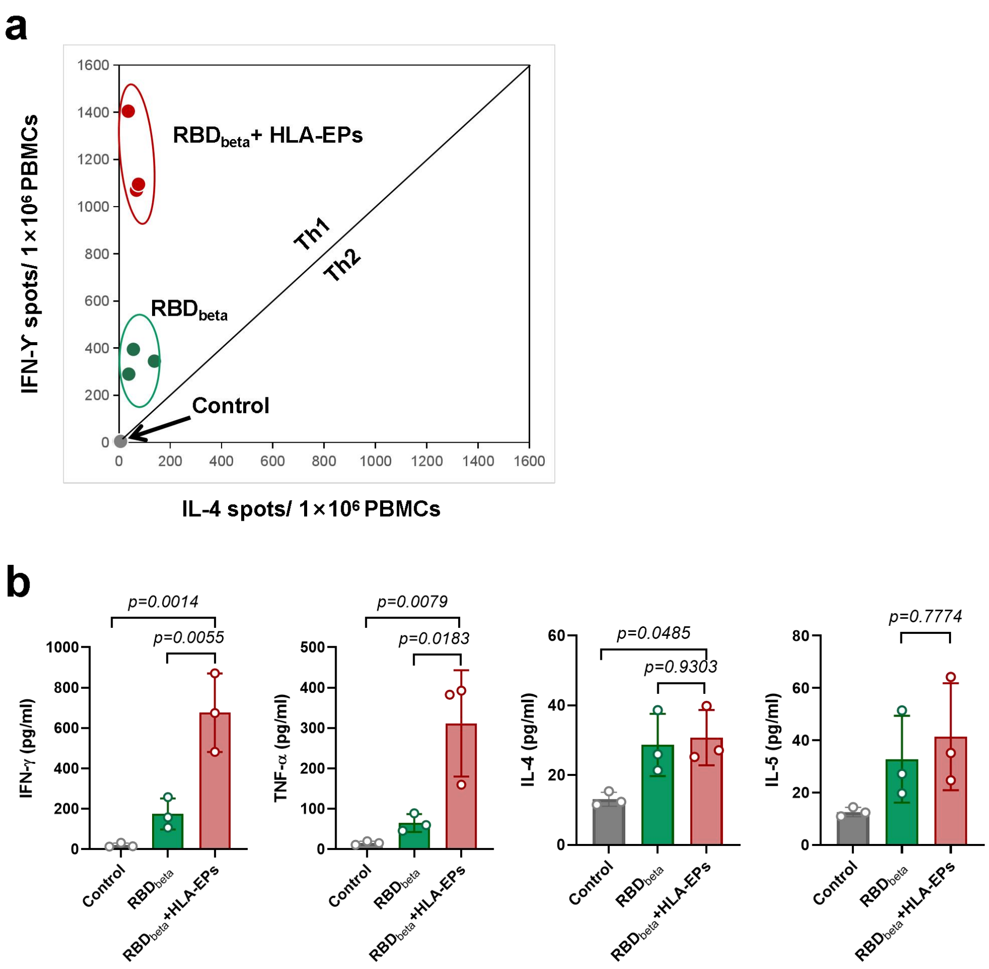

Supplementary Fig. 7 Induction of Th1-biased cellular immune response by dual immunization in macaques.

Female macaques (2-3 years old) were immunized twice intramuscularly with LNP-RBD_{beta}+ LNP-*HLA-EPs* (n = 3) following a full vaccination procedure (two doses with a 3-week interval). Groups of macaques immunized with LNP-RBD_{beta} alone or PBS served as controls. PBMCs were isolated 35 days after the initial vaccination for T-cell response evaluation. **a** Scatter plot showed the correlation of IL-4- and IFN-γ-secreting cells in PBMCs measured by ELISpot (14 days post-booster vaccination). **b** Cytokine production by PBMCs 14 days after the booster immunization was determined by ELISA analysis of IFN-γ, TNF-α, IL-4 and IL-5 in the peptide-stimulated PBMCs. Data from a representative independent experiment of the two are presented. Data points represent the averages of three technical replicates, and their mean ± S.E.M are presented. (n = 3). Source data are provided as a Source Data file. Adjusted *p* values represent comparisons between the groups using one-way ANOVA with Tukey's multiple comparison post-hoc two-sided tests.

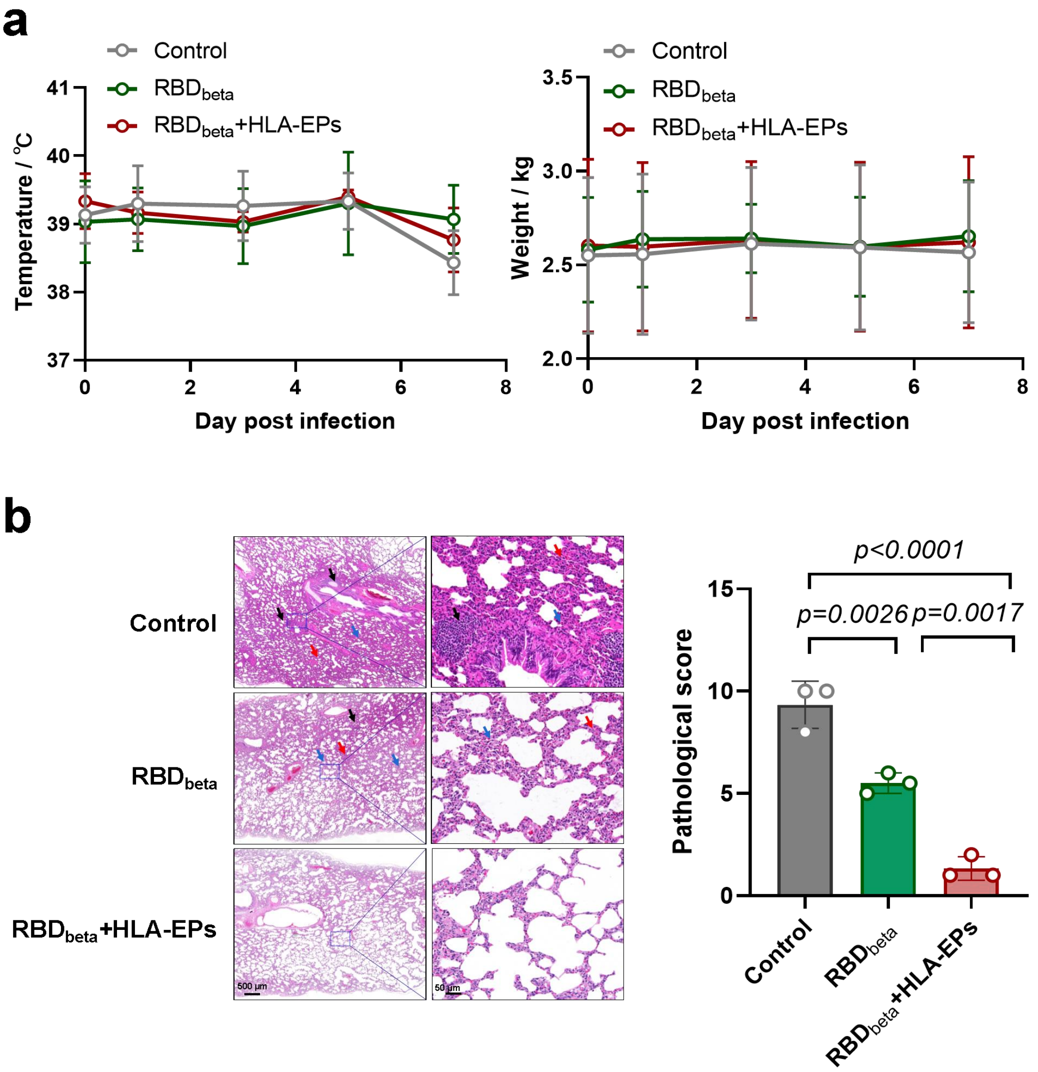

Supplementary Fig. 8 Clinical and pathological signs in immunized macaques after challenge with SARS-CoV-2.

Female macaques (2-3 years old) were immunized twice intramuscularly with LNP-*RBD$_{beta}$*+ LNP-*HLA-EPs* following a full vaccination procedure (two doses with a 3-week interval, n = 3). Groups of macaques immunized with LNP-*RBD$_{beta}$* alone (n = 3) or PBS (n = 3) served as controls. All macaques were challenged with SARS-CoV-2 (B.1.351 variant) at three weeks after the booster vaccination. **a** Both temperatures (upper panel) and weights (lower panel) were recorded on Day 0, 1, 3, 5, and 7 post-challenge. The data are presented as the mean ± S.E.M. (n = 3). Source data are provided as a Source Data file. **b** Histopathological changes in the lung tissues collected 7 days post-live virus challenge were evaluated by H&E staining. H&E-stained lung tissue sections were blindly examined and scored by trained histo-pathologists, and the data are presented as the mean ± S.E.M. (n = 3). Images derived

from one representative animal in each group with sites of inflammatory cell infiltration (black arrows), blood clots (blue arrows), and alveolar deformation (red arrows) are presented. Scale bar is 500 μm for the lower magnification and 50 μm. Five sections were taken from each macaque for histopathology analysis. Source data are provided as a Source Data file. Adjusted *p* values represent comparisons between groups using one-way ANOVA with Tukey's multiple comparison post-hoc two-sided tests (**b**).

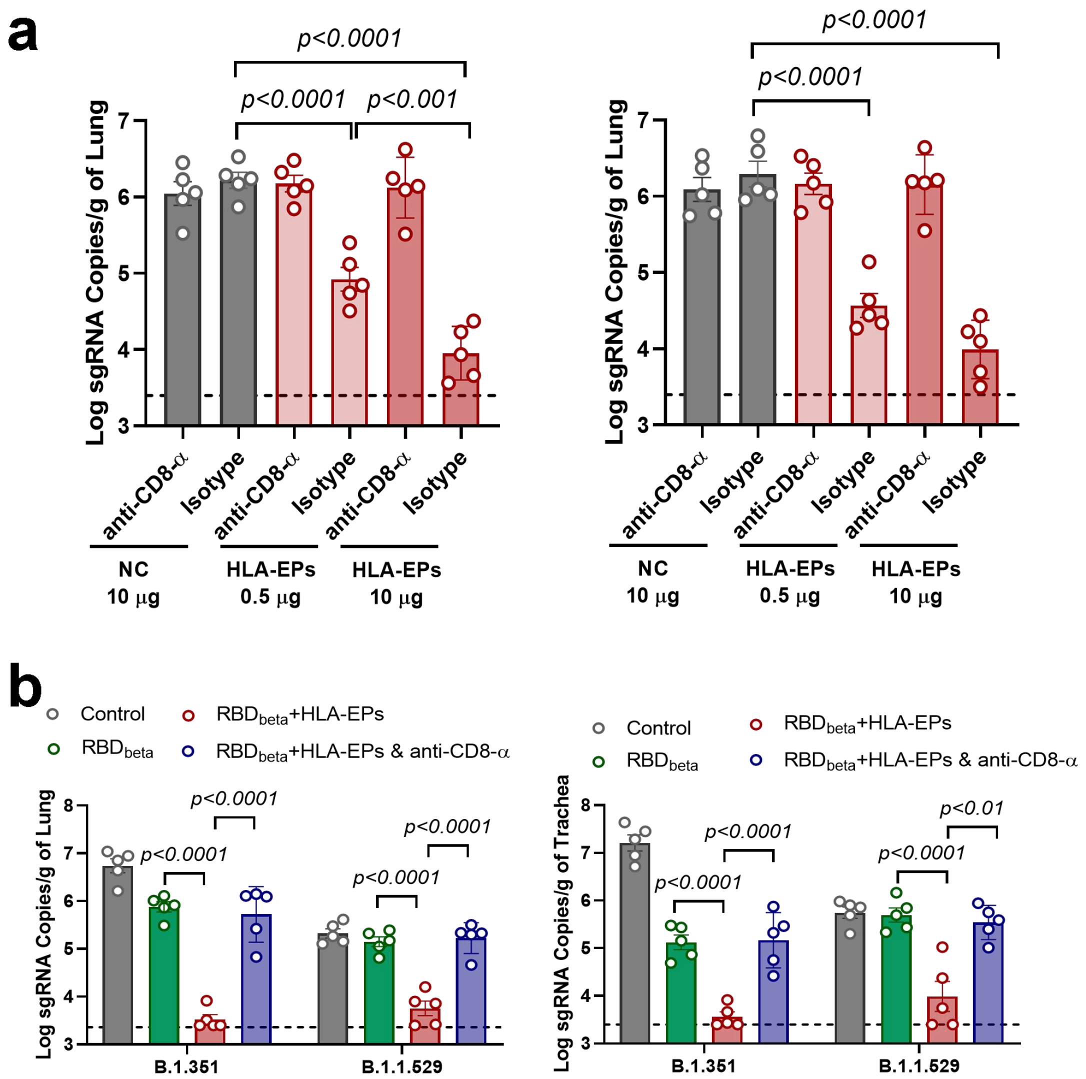

Supplementary Fig. 9 Viral load in tissues from challenged animals by qRT-PCR.

a $CD8^+$ T-cell dependent protective efficacy of LNP-*HLA-EPs* against SARS-CoV-2 infection in HLA-transgenic mice. Both HLA-A*02:01/DR1 and HLA-A*11:01/DR1 transgenic mice were inoculated with either a low (0.5 μg) or a high (10 μg) doses of LNP-*HLA-EPs* or LNP-*NC* (10 μg) following a full vaccination procedure (two doses with a 3-week interval). Twenty days after the booster immunization, the mice under all vaccination schemes were randomly divided into two subgroups and then inoculated twice with either an anti-CD8-α antibody to deplete $CD8^+$ T cells or an isotype IgG2b control. The mice were then infected with the SARS-CoV-2 B.1.351 variant (1.4 × 10^5 PFU/mouse). The viral loads in the lungs were quantitated by qRT–PCR at 4 days post-infection. Left panel, HLA-A*02:01 mice. Right panel, HLA-A*11:01 mice. **b** The protective efficacy of dual immunization against infection of the SARS-CoV-2 B.1.351 strain and B.1.1.529 variant in HLA-A*02:01 transgenic mice.

HLA-A*02:01/DR1 HLA-transgenic mice were immunized by the combination of 0.5 μg LNP-*HLA-EPs* and 0.5 μg LNP-*NC* following a full vaccination procedure (two doses with a 3-week interval). The animals inoculated with 0.5 μg LNP-RBD_{beta} and PBS served as controls. Twenty-one days after the booster vaccination, the immunized mice were challenged with either the SARS-CoV-2 B.1.351 variant (1.4×10^5 PFU/mouse) or B.1.1.529 variant (2.0×10^5 PFU/mouse). The viral loads in the lungs (left panel) and tracheae (right panel) were assessed at 4 days after the viral challenge. The result shown was representative of two independent experiments with 2 (**a,b**). Data are presented as the mean ± S.E.M. of 5 (mouse) or 3 (macaques) biological replicates. Source data are provided as a Source Data file. Adjusted *p* values were calculated via two-way ANOVA with Bonferroni's multiple comparison post-hoc two-sided tests (**a**) or one-way ANOVA with Tukey's multiple comparisons (**b**).

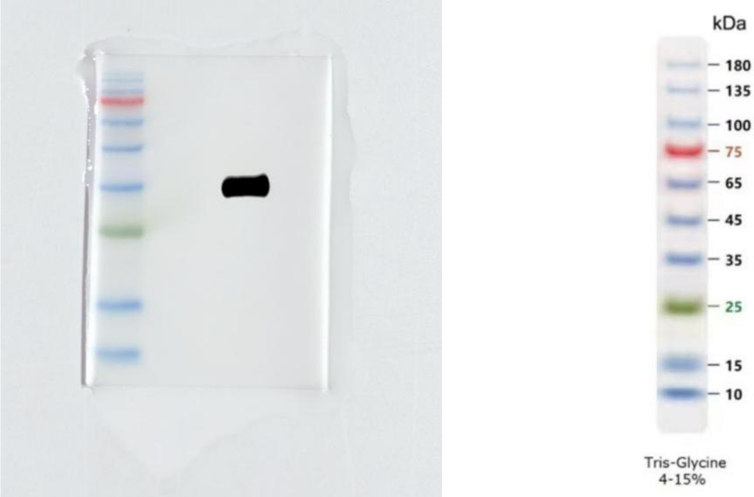

Supplementary Fig. 10 Uncropped blots for Supplementary Fig. 4b. The molecular weights (kDa) of protein markers are listed on the right, and the image is representative of the similar results from two independent experiments.

Supplementary Table 1 The HLA-I alleles selected for functional epitopes prediction.

HLA-I alleles				
**A alleles**	**B alleles**	**C alleles**	**E alleles**	**G alleles**
A*01:01	B*07:02	C*01:02	E*01:01	G*01:01
A*02:01	B*08:01	C*02:02	E*01:03	G*01:02
A*02:06	B*13:01	C*02:09		G*01:03
A*03:01	B*13:02	C*03:02		G*01:04
A*11:01	B*14:02	C*03:03		G*01:06
A*23:01	B*15:01	C*03:04		
A*24:02	B*15:02	C*04:01		
A*25:01	B*15:25	C*05:01		
A*26:01	B*18:01	C*06:02		
A*29:02	B*27:02	C*07:01		
A*30:01	B*27:05	C*07:02		
A*30:02	B*35:01	C*07:04		
A*31:01	B*35:03	C*08:01		
A*32:01	B*37:01	C*08:02		
A*33:01	B*38:01	C*12:02		
A*33:03	B*39:01	C*12:03		
A*68:01	B*40:01	C*14:02		
A*68:02	B*40:02	C*15:02		
A*74:01	B*44:02	C*16:01		
	B*44:03	C*17:01		
	B*46:01			
	B*48:01			
	B*49:01			
	B*50:01			
	B*51:01			
	B*52:01			
	B*53:01			
	B*55:01			
	B*56:01			
	B*57:01			
	B*58:01			
	B*58:02			

Supplementary Table 2 PBMCs donors information.

Donor No.	sex	Age range	HLA-I alleles		Days post recovery
1	M	40-49	A*02:01	A*32:01	139
2	F	30-39	A*02:01	A*02:06	242
3	F	30-39	A*02:01	A*02:01	192
4	F	20-29	A*02:01	A*11:01	195
5	M	20-29	A*02:01	A*24:02	134
6	M	20-29	A*02:01	A*11:01	145
7	M	40-49	A*02:01	A*11:01	90
8	F	30-39	A*02:01	A*03:01	82
9	F	40-49	A*02:01	A*33:01	95
10	F	40-49	A*11:01	A*32:01	90
11	F	30-39	A*11:01	A*02:06	152
12	M	20-29	A*11:01	A*02:06	166
13	M	40-49	A*11:01	A*24:02	109
14	M	30-39	A*11:01	A*02:01	93
15	F	30-39	A*11:01	A*24:02	150

M, male; F, female

Supplementary Table 3 Predicted epitopes mutation summary of NSP-4$_{232-444}$ and NSP-6$_{1-201}$.

Identity	Variant	Mutant Position	Epitope	Affinity IC50 (nM)	HLA Allele
NSP-4$_{232-444}$	WT	T327	FTVLCLTPV	4.65	A*02:06
NSP-4$_{232-444}$	BA.2	T327I	FIVLCLTPV	5.77	A*02:06
NSP-6$_{1-201}$	WT	I189	FLARGIVFM	6.13/ 5.73	A*02:01/ A*02:03
NSP-6$_{1-201}$	B.1.1.529 (BA.1)	I189V	FLARGVVFM	6.68/ 6.11	A*02:01/ A*02:03
NSP-6$_{1-201}$	WT	T77	FLCLFLLPSLATV	8.7/ 8.88	A*02:01/ A*02:03
NSP-6$_{1-201}$	B.1.617.2 (Delta)	T77A	FLCLFLLPSLAAV	8.33/ 7.74	A*02:01/ A*02:03
NSP-6$_{1-201}$	WT	T77	FLLPSLATV	2.41/ 2.18/ 2.46	A*02:01/ A*02:03/ A*02:06
NSP-6$_{1-201}$	B.1.617.2 (Delta)	T77A	FLLPSLAAV	2.15/ 1.93/ 2.14	A*02:01/ A*02:03/ A*02:06
NSP-6$_{1-201}$	WT	T77	LFLLPSLATV	6.08/ 4.95/ 9.73	A*02:01/ A*02:03/ A*02:06
NSP-6$_{1-201}$	B.1.617.2 (Delta)	T77A	LFLLPSLAAV	5.54/ 4.32/ 8.04	A*02:01/ A*02:03/ A*02:06
NSP-6$_{1-201}$	WT	T77	FLLPSLATVA	6.84/ 5.38	A*02:01/ A*02:03
NSP-6$_{1-201}$	B.1.617.2 (Delta)	T77A	FLLPSLAAVA	6.29/ 4.75	A*02:01/ A*02:03
NSP-6$_{1-201}$	WT	T77	LPSLATVAY	2.6/ 5.38	B*35:01/ A*02:03
NSP-6$_{1-201}$	B.1.617.2 (Delta)	T77A	LPSLAAVAY	3.12/ 4.75	B*35:01/ A*02:03
NSP-6$_{1-201}$	WT	T77	LPSLATVAY	2.6	B*35:01
NSP-6$_{1-201}$	B.1.617.2 (Delta)	T77A	LPSLAAVAY	3.12	B*35:01
NSP-6$_{1-201}$	WT	T77	ATVAYFNMV	8.76	A*02:06
NSP-6$_{1-201}$	B.1.617.2 (Delta)	T77A	AAVAYFNMV	16.34	A*02:06
NSP-6$_{1-201}$	WT	T77	TVAYFNMVY	6.01	A*29:06
NSP-6$_{1-201}$	B.1.617.2 (Delta)	T77A	AVAYFNMVY	8.06	A*29:06

Supplementary Table 4 Validated HLA-A*02:01 and HLA-A*11:01 peptides in tetramer assay.

Peptide	Position	HLA-I Alleles	Variant	Affinity IC50 (nM)	Reference
FLLNKEMYL	NSP-4$_{420-428}$	HLA-A*02:01	WT	2.67	69
SMWALIISV	NSP-6$_{109-117}$	HLA-A*02:01	WT	3.80	70
ALCTFLLNK	NSP-4$_{416-424}$	HLA-A*11:01	WT	19.49	38
SAFAMMFVK	NSP-6$_{53-61}$	HLA-A*11:01	WT	5.75	48
LTNMFTPLI	NSP-4$_{264-272}$	HLA A*02:01	WT	16.66	this paper
FTNMFTPLI	NSP-4$_{264-272}$	HLA A*02:01	B.1.1.7	103.73	this paper
TSLSGFKLK	NSP-6$_{103-111}$	HLA A*11:01	WT	22.93	this paper
MVDTSLKLK	NSP-6$_{100-108}$	HLA A*11:01	B.1.351/P.1	29.39	this paper
MVDTSFKLK	NSP-6$_{100-108}$	HLA A*11:01	B.1.1.529	50.38	this paper
VTTVMVLAR	NSP-6$_{179-187}$	HLA A*11:01	WT	167.46	this paper
VTTVMFLAR	NSP-6$_{179-187}$	HLA A*11:01	P.1	226.17	this paper
FLARGIVFM	NSP-6$_{184-192}$	HLA A*02:01	WT	6.13	this paper
VLARGVVFM	NSP-6$_{184-192}$	HLA A*02:01	P.1	70.56	this paper
FLARGIVFM	NSP-6$_{184-192}$	HLA A*02:01	B.1.1.529	6.68	this paper
TLMNVLTLA	NSP-6$_{141-149}$	HLA A*02:01	WT	3.31	this paper
TLMNVLTLV	NSP-6$_{141-149}$	HLA A*02:01	P.1	8.41	this paper
NVLTLAYKV	NSP-6$_{144-152}$	HLA A*02:01	WT	73.8	this paper
NVLTLVYKV	NSP-6$_{144-152}$	HLA A*02:01	P.1	287.24	this paper

Supplementary Table 5 Primers for qRT–PCR.

Primer	Sequence
CXCL13-F	5′-TCTGGAAGCCCATTACACAA-3′
CXCL13-R	5′-TTTGTAACCATTTGGCACGA-3′
CXCR5-F	5′-GGTGCTGGTAATCCTGGAGA-3′
CXCR5-R	5′-GATCTTGTGCAGAGCGATCA-3′
SARS-CoV-2 sgRNA-F	5′-CGATCTCTTGTAGATCTGTTCTC-3′
SARS-CoV-2 sgRNA-R	5′-ATATTGCAGCAGTACGCACACA-3′
SARS-CoV-2 sgRNA-Probe	5'-FAM-ACACTAGCCATCCTTACTGCGCTTCG-BHQ1-3'
mouse GAPDH-F	5′-TCAACAGCAACTCCCACTCTTCCA-3′
mouse GAPDH-R	5′-ACCCTGTTGCTGTAGCCGTATTCA-3′
