# Supplementary material for: An mRNA-based T-cell-inducing antigen strengthens COVID-19 vaccine against SARS-CoV-2 variants
Source: Nat Commun. 2023 May 23;14:2962. doi: 10.1038/s41467-023-38751-8 (PMC10204679; doi:10.1038/s41467-023-38751-8)
Supplement: Supplementary file 3 — Description of Additional Supplementary Files [file 41467_2023_38751_MOESM3_ESM.pdf]

Description of Additional Supplementary Files

File Name: Supplementary Data 1
Description: Predicted effective epitopes in NSP-31443-1605, NSP-4232-444, NSP-61-201, M1-113, NSP-11-180, NSP-31066-1278, NSP-14330-490, and NFull-length.
