# Supplementary material for: An mRNA-based T-cell-inducing antigen strengthens COVID-19 vaccine against SARS-CoV-2 variants
Source: Nat Commun. 2023 May 23;14:2962. doi: 10.1038/s41467-023-38751-8 (PMC10204679; doi:10.1038/s41467-023-38751-8)
Supplement: Supplementary file 5 — Reporting Summary [file 41467_2023_38751_MOESM5_ESM.pdf]

nature portfolio

Corresponding author(s): Gong Cheng

Last updated by author(s): May 5, 2023

Reporting Summary

Nature Portfolio wishes to improve the reproducibility of the work that we publish. This form provides structure for consistency and transparency in reporting. For further information on Nature Portfolio policies, see our Editorial Policies and the Editorial Policy Checklist.

Statistics

For all statistical analyses, confirm that the following items are present in the figure legend, table legend, main text, or Methods section.

n/a	Confirmed	
☐	☒	The exact sample size (*n*) for each experimental group/condition, given as a discrete number and unit of measurement
☐	☒	A statement on whether measurements were taken from distinct samples or whether the same sample was measured repeatedly
☐	☒	The statistical test(s) used AND whether they are one- or two-sided<br>*Only common tests should be described solely by name; describe more complex techniques in the Methods section.*
☒	☐	A description of all covariates tested
☒	☐	A description of any assumptions or corrections, such as tests of normality and adjustment for multiple comparisons
☐	☒	A full description of the statistical parameters including central tendency (e.g. means) or other basic estimates (e.g. regression coefficient) AND variation (e.g. standard deviation) or associated estimates of uncertainty (e.g. confidence intervals)
☐	☒	For null hypothesis testing, the test statistic (e.g. *F*, *t*, *r*) with confidence intervals, effect sizes, degrees of freedom and *P* value noted<br>*Give P values as exact values whenever suitable.*
☒	☐	For Bayesian analysis, information on the choice of priors and Markov chain Monte Carlo settings
☒	☐	For hierarchical and complex designs, identification of the appropriate level for tests and full reporting of outcomes
☒	☐	Estimates of effect sizes (e.g. Cohen's *d*, Pearson's *r*), indicating how they were calculated

Our web collection on statistics for biologists contains articles on many of the points above.

Software and code

Policy information about availability of computer code

Data collection	IEDB server (TepiTool (iedb.org) V2.22), NetMHCpan 4.1 server (NetMHCpan 4.1 - DTU Health Tech - Bioinformatic Services), BD Aria III cell analyzer (BD FACSAriaTM III), BD LSRFortessa (BD LSRFortessaTM), Dynamic light scattering (Dynapro NanoStar, Wyatt), IVIS Spectrum In Vivo Imaging System (Lumina II, PerkinElmer), Varioskan Flash microplate reader (WARIOSKAN FLASH, Thermo Scientific), ELISpot reader (iSpot, AID).
Data analysis	Graphpad Prism 8.0, Living Image 4.5, FlowJo software V_10.

For manuscripts utilizing custom algorithms or software that are central to the research but not yet described in published literature, software must be made available to editors and reviewers. We strongly encourage code deposition in a community repository (e.g. GitHub). See the Nature Portfolio guidelines for submitting code & software for further information.

Data

Policy information about availability of data

All manuscripts must include a data availability statement. This statement should provide the following information, where applicable:

- Accession codes, unique identifiers, or web links for publicly available datasets
- A description of any restrictions on data availability
- For clinical datasets or third party data, please ensure that the statement adheres to our policy

Data generated and analyzed in this study are provided in the Source Data file. The HLA class I allele information listed is based on structural and nonstructural proteins of the SARS-CoV-2 Wuhan-Hu-1 (wild-type) strain (Accession number: NC_045512.2).

Human research participants

Policy information about studies involving human research participants and Sex and Gender in Research.

Reporting on sex and gender	We included 7 male and 8 female (biological attribute). Detailed Information about sex (based on the assigned documents) of participants has been provided in Supplementary Table 2.
Population characteristics	We enrolled 15 participants with the ages between 20 and 50. Detailed information of the population is provided in Supplementary Table 2.
Recruitment	A cohort of 15 convalescent COVID-19 donors were recruited for blood donation (Supplementary Table 2). Eligible participants were people 18 to 65 years of age, and key exclusion criteria included previous vaccination with any coronavirus vaccine, known acute and chronic infectious diseases, severe chronic and bleeding disorders, pregnancy, and lactation. Participants were recruited using digital advertising strategies. No potential selection bias was present.
Ethics oversight	The protocol for PBMC collection was approved by the biomedical ethical committee of Wenzhou Central Hospital (K2020-01-005 (5)).

Note that full information on the approval of the study protocol must also be provided in the manuscript.

Field-specific reporting

Please select the one below that is the best fit for your research. If you are not sure, read the appropriate sections before making your selection.

☒ Life sciences ☐ Behavioural & social sciences ☐ Ecological, evolutionary & environmental sciences

For a reference copy of the document with all sections, see nature.com/documents/nr-reporting-summary-flat.pdf

Life sciences study design

All studies must disclose on these points even when the disclosure is negative.

Sample size	Blood samples were obtained from 15 participants and all samples collected were analyzed. The sample size of could reach statistical significance of CD8+ T cells activation percentages by one-way ANOVA with Tukey's multiple comparison post-hoc two-sided test. Mice number (n=5, each group) was selected to maintain the animals number n>4/group throughout the study period considering the interim tissue collection (Dinon, et al. Nature. 2020, 586, 560-566.). Macaques number (n=3, each group) was selected based on previous publications (Salguero, et al. Nat Commun. 2021, 12, 1260; McMahan, et al. Nature. 2021, 590, 630-634.). No statistical methods were used to predetermine the sample size.
Data exclusions	No data were excluded from the analyses.
Replication	Experimental assays were performed in duplicate (or more) according to or exceeding standards in the field. All attempts at replication were successful. In all cases, representative figure displays were appropriately indicated.
Randomization	Animals were randomly allocated to the groups. For other experiments, randomization was not a relevant feature as we were applying a uniform set of techniques.
Blinding	The lung pathology evaluation was run blindly by the investigators. For other experiments, blinding was not relevant as this is an observational study and we were applying a uniform set of measurements.

Reporting for specific materials, systems and methods

We require information from authors about some types of materials, experimental systems and methods used in many studies. Here, indicate whether each material, system or method listed is relevant to your study. If you are not sure if a list item applies to your research, read the appropriate section before selecting a response.

Materials & experimental systems

n/a	Involved in the study
☐	☒ Antibodies
☐	☒ Eukaryotic cell lines
☒	☐ Palaeontology and archaeology
☐	☒ Animals and other organisms
☒	☐ Clinical data
☒	☐ Dual use research of concern

Methods

n/a	Involved in the study
☒	☐ ChIP-seq
☐	☒ Flow cytometry
☒	☐ MRI-based neuroimaging

Antibodies

Antibodies used

anti-SARS-CoV-2 S1 antibody (Supplier: Sino Biological, Cat#: 40150-R007, clone: #007), Western blot: 1:1000 dilution.
HRP-conjugated goat anti-rabbit antibody (Supplier: Invitrogen, Cat#: 32460), Western blot: 1:3000 dilution.
anti-Flag tag rabbit mAb (Supplier: Cell Signaling Technology, Cat#: 14793S, clone: D6W5B), flow cytometry: 1:1000 dilution.
goat anti-rabbit IgG H&L (Alexa Fluor® 555) (Supplier: Abcam, Cat#: ab150078), flow cytometry: 1:500 dilution.
anti-V5 tag mouse mAb (Supplier: Cell Signaling Technology, Cat#: 80076S, clone: E9H8O), flow cytometry: 1:1000 dilution.
goat anti-mouse IgG H&L (Cy3®) (Supplier: Abcam, Cat#: ab97035), flow cytometry: 1:1000 dilution.
HRP-conjugated anti-mouse IgG antibodies (Supplier: Invitrogen, Cat#: A16066), ELISA: 1:5000 dilution.
HRP-conjugated anti-monkey IgG antibodies (Supplier: Invitrogen, Cat#: PA1-84631), ELISA: 1:5000 dilution.
anti-CD45-Alexa Fluor™ 700 (Supplier: Invitrogen, Cat#: 56-0451-82, clone: 30-F11), flow cytometry: 0.5 μg/test, 1:40 dilution.
anti-CD4-FITC (Supplier: Tonbo Biosciences, Cat#: 35-0042-U100, clone: RM4-5), flow cytometry: 1 μg/test, 1:50 dilution.
anti-CD185-Brilliant Violet 605™ (Supplier: BioLegend, Cat#: 145513, clone: L138D7), flow cytometry: 5 μl/test, 1:25 dilution.
anti-PD-1-Brilliant Violet 421™ (Supplier: BioLegend, Cat#: 135218, clone: 29F.1A12), flow cytometry: 0.125 μg/test, 1:20 dilution.
anti-B220-PerCP-Cyanine5.5 (Supplier: TONBO Biosciences, Cat#: 65-0452-U100, clone: RA3-6B2), flow cytometry: 1 μg/test, 1:20 dilution.
anti-CD95-PE (Supplier: BioLegend, Cat#: 152608, clone: SA367H8), flow cytometry: 0.125 μg/test, 1:160 dilution.
anti-GL-7-APC (Supplier: BioLegend, Cat#: 144618, clone: GL7), flow cytometry: 0.25 μg/test, 1:80 dilution.
anti-CD8-PerCP-Cyanine5.5 (Supplier: TONBO Biosciences, Cat#: 65-0081-U100, clone: 53-6.7), flow cytometry: 1 μg/test, 1:20 dilution.
anti-CD44-APC (Supplier: BioLegend, Cat#: 103012, clone: IM7), flow cytometry: 0.25 μg/test, 1:80 dilution.
anti-CD62L-BV421 (Supplier: BioLegend, Cat#: 104436, clone: MEL-14), flow cytometry: 5 μl/test, 1:20 dilution.
anti-IFN-γ-PE (Supplier: TONBO Biosciences, Cat#: 50-7311-U100, clone: XMG1.2), flow cytometry: 1 μg/test, 1:20 dilution.
anti-TNF-α-BV421 (BioLegend, Cat#: 506328, clone: MP6-XT22) antibody, flow cytometry: 0.25 μg/test, 1:80 dilution.

Validation

All antibodies are commercially available and were commercially validated.
anti-SARS-CoV-2 S1 antibody (Supplier: Sino Biological, Cat#: 40150-R007, clone: #007) was validated by successful ELISA analysis according to the manufacturer's website https://www.sinobiological.com/antibodies/cov-spike-40150-r007.
HRP-conjugated goat anti-rabbit antibody (Supplier: Invitrogen, Cat#: 32460) was validated by successful staining analysis according to the manufacturer's website https://www.thermofisher.com/antibody/product/Goat-anti-Rabbit-IgG-H-L-Secondary-Antibody-Polyclonal/32460.
anti-Flag tag rabbit mAb (Supplier: Cell Signaling Technology, Cat#: 14793S, clone: D6W5B) was validated by successful staining analysis according to the manufacturer's website https://www.cellsignal.cn/products/primary-antibodies/dykddddk-tag-d6w5b-rabbit-mab-binds-to-same-epitope-as-sigma-s-anti-flag-m2-antibody/14793.
goat anti-rabbit IgG H&L (Alexa Fluor® 555) (Supplier: Abcam, Cat#: ab150078) was validated by successful staining analysis according to the manufacturer's website https://www.abcam.com/products/secondary-antibodies/goat-rabbit-igg-hl-alexa-fluor-555-ab150078.html.
anti-V5 tag mouse mAb (Supplier: Cell Signaling Technology, Cat#: 80076S, clone: E9H8O) was validated by successful staining analysis according to the manufacturer's website https://www.cellsignal.com/products/primary-antibodies/v5-tag-e9h8o-mouse-mab/80076.
goat anti-mouse IgG H&L (Cy3®) (Supplier: Abcam, Cat#: ab97035) was validated by successful staining analysis according to the manufacturer's website https://www.abcam.com/products/secondary-antibodies/goat-mouse-igg-hl-cy3--preadsorbed-ab97035.html.
HRP-conjugated anti-mouse IgG antibodies (Supplier: Invitrogen, Cat#: A16066) was validated by successful ELISA analysis according to the manufacturer's website https://www.thermofisher.com/antibody/product/Goat-anti-Mouse-IgG-H-L-Secondary-Antibody-Polyclonal/A16066.
HRP-conjugated anti-monkey IgG antibodies (Supplier: Invitrogen, Cat#: PA1-84631) was validated by successful ELISA analysis according to the manufacturer's website https://www.thermofisher.com/antibody/product/Goat-anti-Monkey-IgG-H-L-Secondary-Antibody-Polyclonal/PA1-84631.
anti-CD45-Alexa Fluor™ 700 (Supplier: Invitrogen, Cat#: 56-0451-82, clone: 30-F11) was validated by successful flow cytometry analysis according to the manufacturer's website https://www.thermofisher.com/antibody/product/CD45-Antibody-clone-30-F11-Monoclonal/56-0451-82.
anti-CD4-FITC (Supplier: Tonbo Biosciences, Cat#: 35-0042-U100, clone: RM4-5) was validated by successful flow cytometry analysis according to the manufacturer's website https://www.hoelzel-biotech.com/media/import/pdf_pds/Tonbo_Biosciences/35-0042-U100__PDS.pdf.
anti-CD185-Brilliant Violet 605™ (Supplier: BioLegend, Cat#: 145513, clone: L138D7) was validated by successful flow cytometry analysis according to the manufacturer's website 5 μl/test. https://www.biolegend.com/en-us/products/brilliant-violet-605-anti-mouse-cd185-cxcr5-antibody-8616?GroupID=BLG11476.

anti-PD-1-Brilliant Violet 421™ (Supplier: BioLegend, Cat#: 135218, clone: 29F.1A12) was validated by successful flow cytometry analysis according to the manufacturer's website https://www.biolegend.com/en-us/products/brilliant-violet-421-anti-mouse-cd279-pd-1-antibody-7330?GroupID=BLG7927.
anti-B220-PerCP-Cyanine5.5 (Supplier: TONBO Biosciences, Cat#: 65-0452-U100, clone: RA3-6B2) was validated by successful flow cytometry analysis according to the manufacturer's website https://cytekbio.com/products/percp-cyanine5-5-anti-human-mouse-cd45r-b220-ra3-6b2?variant=40581197037604.
anti-CD95-PE (Supplier: BioLegend, Cat#: 152608, clone: SA367H8) was validated by successful flow cytometry analysis according to the manufacturer's website https://www.biolegend.com/en-us/products/pe-anti-mouse-cd95-fas-antibody-13907?GroupID=BLG15543.
anti-GL-7-APC (Supplier: BioLegend, Cat#: 144618, clone: GL7) was validated by successful flow cytometry analysis according to the manufacturer's website https://www.biolegend.com/en-us/products/apc-anti-mouse-human-gl7-antigen-t-and-b-cell-activation-marker-antibody-17248.
anti-CD8-PerCP-Cyanine5.5 (Supplier: TONBO Biosciences, Cat#: 65-0081-U100, clone: 53-6.7) was validated by successful flow cytometry analysis according to the manufacturer's website https://cytekbio.com/products/percp-cyanine5-5-anti-mouse-cd8a-53-6-7?variant=40581194711076.
anti-CD44-APC (Supplier: BioLegend, Cat#: 103012, clone: IM7) was validated by successful flow cytometry analysis according to the manufacturer's website https://www.biolegend.com/en-us/products/apc-anti-mouse-human-cd44-antibody-312.
anti-CD62L-BV421 (Supplier: BioLegend, Cat#: 104436, clone: MEL-14) was validated by successful flow cytometry analysis according to the manufacturer's website https://www.biolegend.com/en-us/products/brilliant-violet-421-anti-mouse-cd62l-antibody-7164.
anti-IFN-γ-PE (Supplier: TONBO Biosciences, Cat#: 50-7311-U100, clone: XMG1.2) was validated by successful flow cytometry analysis according to the manufacturer's website https://cytekbio.com/products/pe-anti-mouse-ifn-gamma-xmg1-2?variant=40581207293988.
anti-TNF-α-BV421 (BioLegend, Cat#: 506328, clone: MP6-XT22) was validated by successful flow cytometry analysis according to the manufacturer's website https://www.biolegend.com/en-us/search-results?Clone=MP6-XT22.

Eukaryotic cell lines

Policy information about cell lines and Sex and Gender in Research

Cell line source(s)	HEK293T cells (Cat. No# CRL-3216) were from ATCC; Vero E6 cells (Cat. No# CRL-1586) were from ATCC; HEK293T cells expressing human ACE2 (HEK293T/hACE2) were provided by Prof. Qiang Ding at Tsinghua University.
Authentication	None of the cell lines used were authenticated.
Mycoplasma contamination	All cell lines were negative for mycoplasma contamination.
Commonly misidentified lines (See ICLAC register)	No commonly misidentified lines were used.

Animals and other research organisms

Policy information about studies involving animals; ARRIVE guidelines recommended for reporting animal research, and Sex and Gender in Research

Laboratory animals	BALB/c mice (Female, 6-8 weeks, from SPF (Beijing) biotechnology co., LTD), HLA-A*02:01/DR1 transgenic mice (4-5 weeks, male and female) and HLA-A*11:01/DR1 transgenic mice (4-5 weeks, male and female) were obtained from Beijing Institute of Microbiology and Epidemiology. Female rhesus macaques (2-3 years old) were provided by Kunming National High-level Biosafety Primate Research Center. Animals were housed in an negative pressured isolator under 12h light-dark cycles with temperature at 22 °C and humidity set points 50-60%.
Wild animals	Wild animals were not involved in this study.
Reporting on sex	Female or male mice and female rhesus macaques were used in this study. Sex was not considered in study design.
Field-collected samples	Field-collected samples were not involved in this study.
Ethics oversight	All animal care and experimental procedures were approved by the Institutional Animal Care and Use Committee (IACUC) of Shenzhen Bay Laboratory (BACG202101), and National Kunming High-level Biosafety Primate Research Center (DWSP202108 009) in accordance with the relevant guidelines for the protection of animal subjects.

Note that full information on the approval of the study protocol must also be provided in the manuscript.

Flow Cytometry

Plots

Confirm that:

☒ The axis labels state the marker and fluorochrome used (e.g. CD4-FITC).

☒ The axis scales are clearly visible. Include numbers along axes only for bottom left plot of group (a 'group' is an analysis of identical markers).

☒ All plots are contour plots with outliers or pseudocolor plots.

☒ A numerical value for number of cells or percentage (with statistics) is provided.

Methodology

Sample preparation	1. mRNA-transfected HEK293T cells were pretreated with trypsin to generate single cells. After fixation and permeabilization, the cells were stained for analysis.<br>2. The lymph nodes and spleens were collected from immunized animals, and then homogenized into single-cell suspensions using a syringe plunger and passed through a 70 µm cell strainer in complete RPMI 1640 media containing 10% fetal bovine serum. Cells were washed and resuspended in fresh RPMI-10% FBS media for stimulating and/or staining.
Instrument	BD Aria III cell analyzer, BD LSRFortessa.
Software	FlowJo software V_10.
Cell population abundance	No sorting experiment were applied in this study.
Gating strategy	The gating strategies are shown in Main text and Supplementary Materials. For all experiments, the single cells were gated based on FSC-H/FSC-W or SSC-A/SSC-H. For the validation of mRNA expression, the positive cells were gated based on mock cells and validated by staining with either goat anti-rabbit IgG H&L (Alexa Fluor 555) or goat anti-mouse IgG H&L (Cy3). The IFP-positive HEK293T cells were gated based on the mock cells without incubating with memory CD8+ T cells from convalescent COVID-19 donors. The specific HLA-epitopes positive CD8+ T cells were gated by live spleen cells from PBS immunized mice stained by anti-CD45-Alexa Fluor 700, anti-CD8-PerCP-Cyanine5.5 and PE labeled peptide-tetramer. The antigen specific IFN-γ and/or TNF-α producing T cells from immunized mice were gated from spleen cells with co-expression of CD45, CD8/CD4 and IFN-γ/TNF-α . Tfh cells and GC B cells were defined as CD45+CD4+CD185+PD-1+ and CD45+B220+CD95+GL-7+ in lymph nodes, respectively. The CD44+CD62L-CD8+ T cells, CD44+CD62+-CD8+ T cells and CD44-CD62L+CD8+ T cells were gated for effector memory, central memory and naive CD8+ T cells in spleen.

☒ Tick this box to confirm that a figure exemplifying the gating strategy is provided in the Supplementary Information.
